# Supplementary material for: Definitions of digital biomarkers: a systematic mapping of the biomedical literature
Source: BMJ Health Care Inform. 2024 Apr 8;31(1):e100914. doi: 10.1136/bmjhci-2023-100914 (PMC11015196; doi:10.1136/bmjhci-2023-100914)

SUPPLEMENTARY MATERIAL

S1: Flowchart illustrating the literature search and selection process

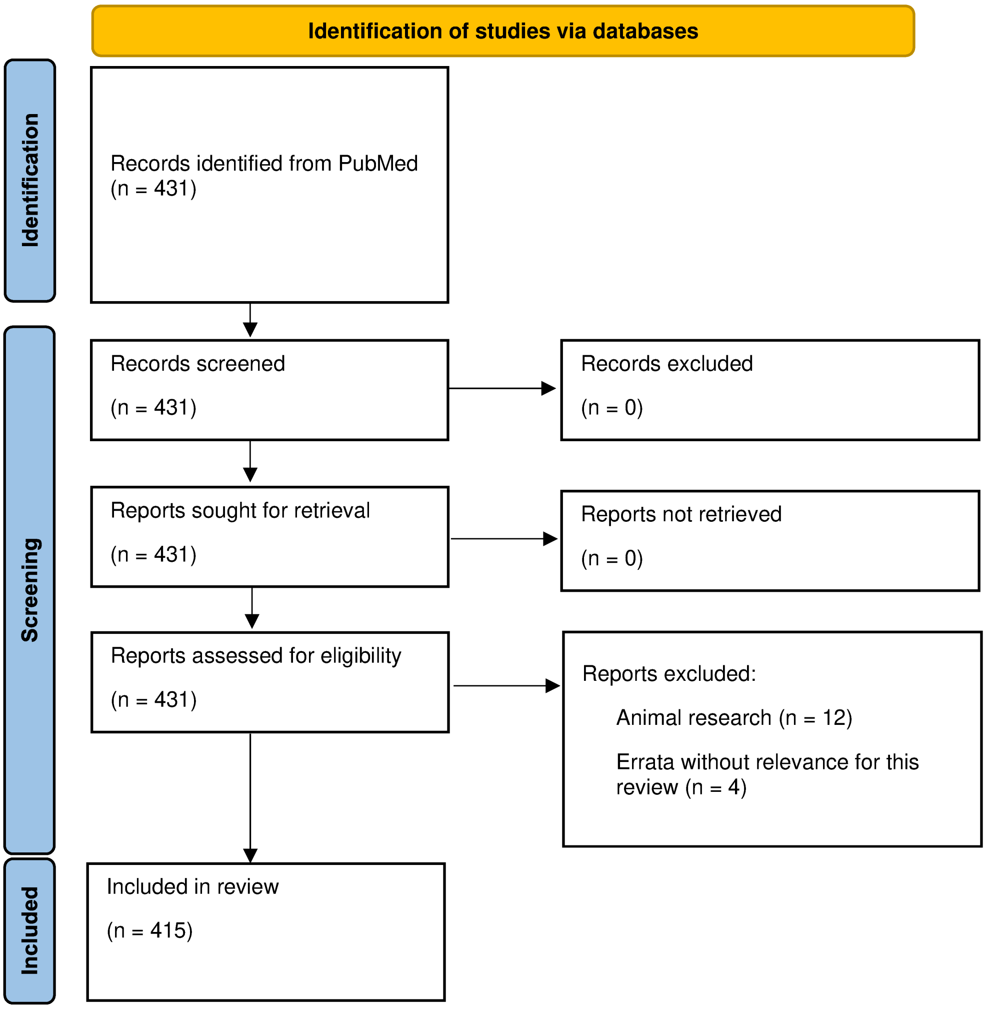

S2. Spreadsheet containing the (sheet 1) bibliography of all identified articles (n=415), (sheet 2) bibliography of all identified articles that provided a definition of digital biomarker (n=128), (sheet 3) characteristics of all identified definitions of digital biomarker (n=202), and (sheet 4) all unique identified definitions of digital biomarker (n=127)

S3. Venn diagram illustrating the components of the identified digital biomarker definitions (n=127)

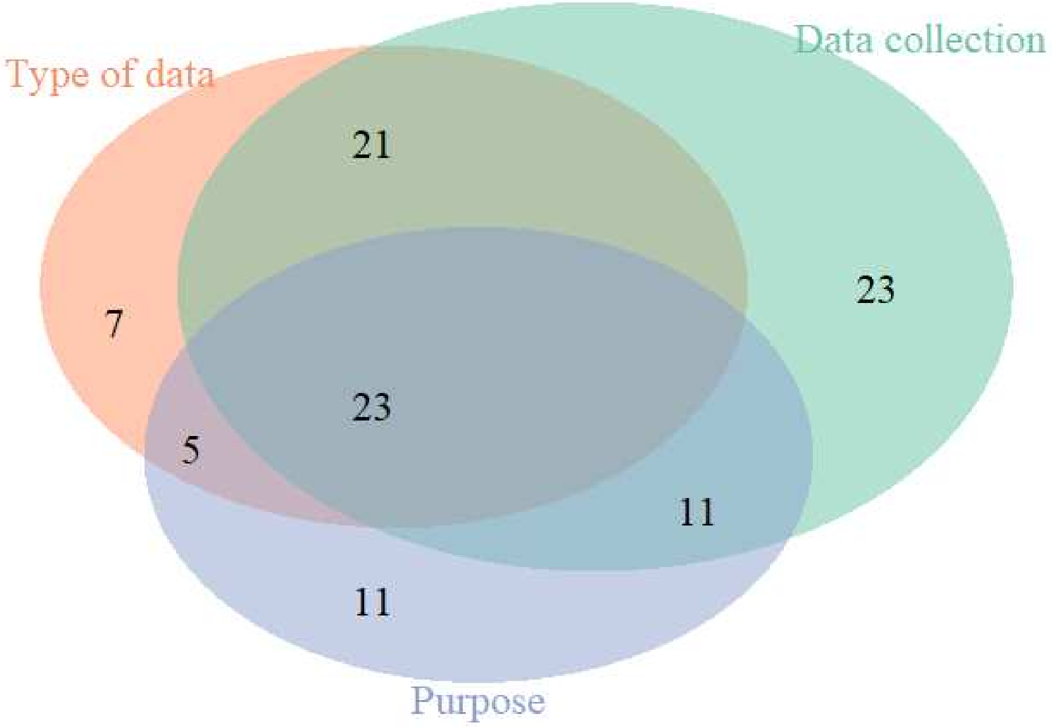

S4. Symmetrical distance-matrix based on the structural Indel-distance of the 51 definitions without a reference (derived from 37 papers). A smaller distance (white / light blue) indicates structurally similar definitions, for which few insertions / deletions are required to change one definition into the other. A larger distance (dark blue) indicates structurally different definitions. The dendrograms at the top and left-hand side are derived through hierarchical-clustering and lead to more similar definitions being clustered next to each other.

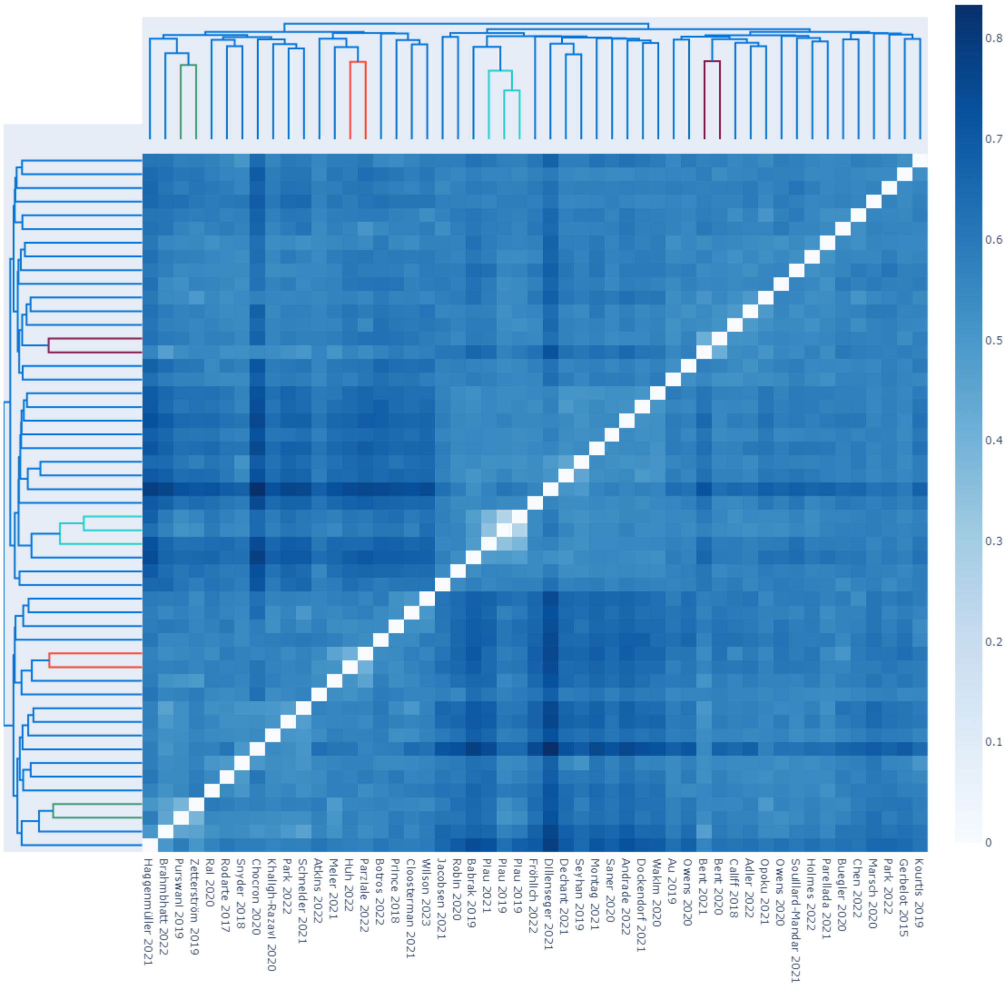

Supplement: Supplementary data [file bmjhci-2023-100914supp001.pdf]
